# Long non-coding RNAs in renal cell carcinoma: A systematic review and clinical implications

**DOI:** 10.18632/oncotarget.17053

**Published:** 2017-04-12

**Authors:** Ming Li, Ying Wang, Liang Cheng, Wanting Niu, Guoan Zhao, Jithin K. Raju, Jun Huo, Bin Wu, Bo Yin, Yongsheng Song, Renge Bu

**Affiliations:** ^1^ Department of Urology, Shengjing Hospital of China Medical University, Shenyang, Liaoning 110004, P.R. China; ^2^ Department of Nuclear Medicine, The First Affiliated Hospital of China Medical University, Shenyang, Liaoning 110001, P.R. China; ^3^ Department of Radiology, Massachusetts General Hospital, Harvard Medical School, Boston, Massachusetts 02114, USA; ^4^ Department of Pathology and Laboratory Medicine, Indiana University School of Medicine, Indianapolis, IN 46202, USA; ^5^ Department of Orthopedics, Brigham and Women's Hospital, VA Boston Healthcare System, Harvard Medical School, Boston, Massachusetts 02115, USA; ^6^ School of Network Education, Beijing University of Posts and Telecommunications, Hebei, Beijing 100088, P.R. China; ^7^ Department of Clinical Medicine, China Medical University, Shenyang, Liaoning 110122, P.R. China

**Keywords:** renal cell carcinoma, lncRNA, diagnosis, prognosis, therapy

## Abstract

Renal cell carcinoma is one of the most common malignancy in adults, its prognosis is poor in an advanced stage and early detection is difficult due to the lack of molecular biomarkers. The identification of novel biomarkers for RCC is an urgent and meaningful project. Long non-coding RNA (lncRNA) is transcribed from genomic regions with a minimum length of 200 bases and limited protein-coding potential. Recently, lncRNAs have been greatly studied in a variety of cancer types. They participate in a wide variety of biological processes including cancer biology. In this review, we provide a new insight of the profiling of lncRNAs in RCC and their roles in renal carcinogenesis, with an emphasize on their potential in diagnosis, prognosis and potential roles in RCC therapy.

## INTRODUCTION

Renal cell carcinoma (RCC) is one of the most common urinary tract malignancies in adults. In 2016, 62,700 newly identified cases and 14240 deaths from kidney and renal pelvis, cancer was estimated to occur in the United States [1]. With mortality at about 3% of all cases and the rate continues to remain very high [1]. In China, an estimated of 66.8 ‰ new cases and 23.4 ‰ deaths from renal cancer occurred in 2015 [2]. The most prevalent RCC histological subtype is clear cell RCC (ccRCC), which accounts for 70% of RCC, followed by papillary RCC (pRCC) and chromophobe RCC (chRCC) (with a prevalence of 10 and 5%, respectively) [3]. In 2016, world health organization (WHO) modified the classification of RCC and added several newly recognized renal tumors [4].

The tumorigenesis of RCC is extremely complex, not only entailing genetic changes but also including dysregulation of epigenetic pathways [5]. Many well-known key signal transduction pathways such as VHL/HIF, PI3K/Akt/mTOR, Raf/MAPK/ERK Jak/Stat, and Wnt-β-catenin pathway have been demonstrated to be involved in the pathogenesis and development of RCC [6–10]. Although great insight into the epigenetics of RCC has been made, for instance, DNA methylation [11], histone modification [12], as well as noncoding RNA [13]. But the underlying epigenetic mechanisms still need to be further studied. Currently, there are no specific and effective molecular biomarkers for RCC. Thus, the identification of novel biomarkers for RCC is extremely urgent and maybe further serve as therapeutic targets for the treatment.

**Figure 1:**
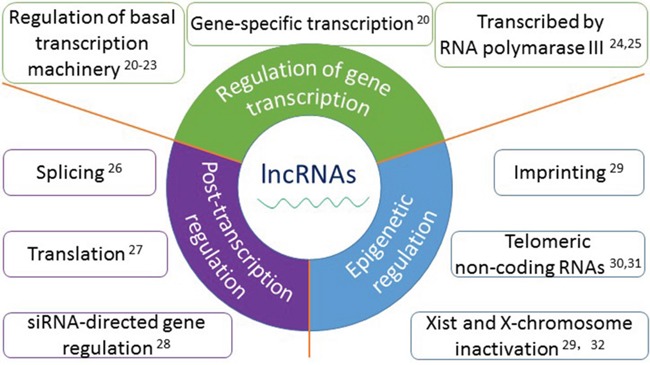
The functions and mechanisms of long non-coding RNAs Accumulated evidence show that lncRNAs play important roles in a wide variety of biological processes, mainly in three aspects: 1. In the regulation of gene transcription, such as regulation of basal transcription machinery, gene-specific transcription and transcribed by RNA polymerase III; 2. In post-transcriptional regulation, such as splicing, translation, and siRNA-directed gene regulation; 3. In epigenetic regulation, such as imprinting, telomeric non-coding RNAs and Xist and X-chromosome inactivation.

It is estimated that 80% of the human genome is transcribed. But only 1-2% of the whole genome is protein-encoded. Remaining non-coding portions of the genome transcription products are non-coding RNAs, they are different in biogenesis, properties and functions [14, 15]. There are many famous non-cording RNAs, microRNAs (miRNAs), small interfering RNAs (siRNAs), transfer RNAs (tRNAs), ribosomal RNAs (rRNAs), small nuclear RNAs (snRNAs), small nucleolar RNAs (snoRNAs), all of which play critical roles in mammalian cells [16]. Most of them are transcribed by RNA polymerase II; some are transcribed by RNA polymerase III [16]. They participate in the epigenetic regulation of other molecules, binding to DNAs, proteins and/or other RNAs [17, 18]. Recently, a newly discovered type of long non-coding RNA molecules (lncRNAs), which are over 200 nucleotides have been greatly studied in multiple diseases and biology [19]. The functions and mechanisms of lncRNAs in mammalian cells are described in detail in Figure 1 [20–32]. Moreover, lncRNAs are involved in cancer biology and have been listed into one of the hallmarks of cancer [33, 34]. They are associated with oncogenesis and may serve as promising a new type of biomarkers for tumor diagnosis, prognosis, even in targeted gene therapy [19, 35, 36].

**Figure 2:**
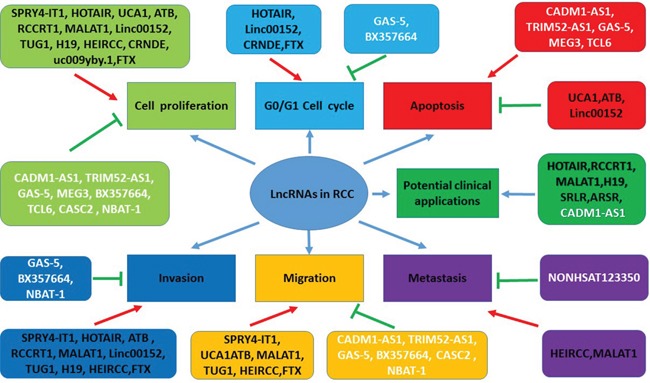
The functions of lncRNAs in pathogenesis and potential clinical applications in RCC LncRNAs play multiple functions in the context of RCC, including regulating cell proliferation, cell cycle, apoptosis, invasion, migration, and metastasis in the forms of oncogenes or tumor suppressor genes. Some lncRNAs maybe serve as therapeutic targets and have the potential of the clinical application. Red arrows indicate promoted signaling pathways. Green arrows indicate inhibited signaling pathways. LncRNAs in black font indicate oncogenes. LncRNAs in white font indicate tumor suppressor genes. Some lncRNAs have multiple functions (for example, MALAT1 promotes cell proliferation, invasion, migration, and metastasis). *Abbreviations: SPRY4-IT1: SPRY4 intronic transcript 1; HOTAIR: Hox transcript antisense intergenic RNA; UCA1: urothelial carcinoma-associated 1; lncRNA-ATB: lncRNA activated by TGF-β; MALAT1: metastasis-associated lung adenocarcinoma transcript 1; Linc00152: long intergenic noncoding RNA 152; TUG1: Taurine up-regulated gene 1; HEIRCC: high-expressed in renal cell carcinoma, lncRNA TCONS_00006756; CRNDE: colorectal neoplasia differentially expressed; CADM1-AS1: lncRNA cell adhesion molecule 1 antisense, lncRNA (RNA176206/ENST00000546273) located in the antisense direction of a coding exon of the cell adhesion molecule1 (CADM1); TRIM52-AS1: TRIM52 antisense RNA 1; GAS-5: growth arrest specific 5; MEG3: maternally expressed gene 3; TCL6: T-cell leukemia/lymphoma 6; CASC2: cancer susceptibility candidate 2; NBAT-1: neuroblastoma associated transcript 1; SRLR: sorafenib resistance-associated lncRNA in RCC; ARSR: lncRNA activated in RCC with sunitinib resistance, ENST00000424980*.

LncRNAs have been found involved in tumorigenesis, disease progression and metastasis of RCC. They function as oncogenes or tumor suppress genes and regulate multiple biologic or pathological processes, which are shown in Figure 2. But the underlying mechanisms still need to be further explored. In this systematic review, we focus on the expression profile of lncRNAs in RCC, with emphasize on the roles of oncogenesis, diagnosis, progression, prognosis and the potential of application in RCC therapy.

## LNCRNAS IN RCC

### Aberrant expression profiling of lncrnas in RCC

Many studies about lncRNAs expression profiling have been carried out in RCC. The determination of their interaction with other molecules and functional analysis is also booming in recent years. The most popular methods for the study of lncRNAs expression profiles of RCC are microarray assay and ChIP-Seq in small sample trials [37–43]. If certain lncRNAs are found to be obviously dysregulated, following qPCR experiments to demonstrate the significance in large scale samples are preferred, some of these significant dysregulated lncRNAs confirmed by qPCR may even serve as a diagnostic or prognostic biomarkers. [37–39]. A couple of recent studies found that hundreds of thousands of lncRNAs were aberrantly expressed in RCC tissue compared with adjacent non-tumor tissue through genome wide assay [37–43]. We summarized these studies in Table 1 in detail [37–43]. In addition, lncRNAs deregulated in RCC are shown in Table 2A and Table 2B; the functions, targeted genes/signaling, and the mechanisms involved are also indicated [44–67].

**Table 1:** The studies about expression profiling of lncRNAs in RCC

Author	Year	Country	RCC subtype	Samplesize (n)	Differentiallyexpressed lncRNAs	Numberupregulated	Numberdownregulated	References
Yu et al	2012	China	ccRCC	6	726	146	480	37
Liu et al	2016	China	not mentioned	90	3862	1649	2243	38
Deng, Blondeau et al	2015	Germany	ccRCC	15	1308	568	740	39, 40
Qin et al	2014	China	ccRCC	5	897	480	417	41
Fachel et al	2013	Brazil	ccRCC	11	40	14	26	42
He et al	2016	China	chRCC	59	143	41	102	43

**Table 2A:** Upregulated lncRNAs in RCC

lncRNAs	Specimens	Functions	Target genes/Signalings	Pathways/Mechanisms involved	Referances
SPRY4-IT1	RCC tissues, cell lines	oncogene		proliferation, migration, invasion	44
HOTAIR	cell lines	oncogene	H3K27me, EZH2, miR-141, Ago2	proliferation, invasion, cell cycle	45, 46
UCA1	RCC tissues, cell lines	oncogene		proliferation, migration, apoptosis	47
lncRNA-ATB	RCC tissues, cell lines	oncogene	EMT	proliferation, apoptosis, migration, invasion	48
RCCRT1	RCC tissues	biomarker		migration, invasion	49
MALAT1	RCC tissues, cell lines	oncogene	Ezh2, miR-205	proliferation, migration, invasion	50, 51
Linc00152	RCC tissues, cell lines	oncogene, biomarker		proliferation, invasion, apoptosis, cell cycle	52
TUG1	RCC tissues, cell lines	oncogene		migration, invasion, proliferation, apoptosis	53
H19	RCC tissues, cell lines	biomarker		proliferation, invasion, migration	54
HEIRCC	RCC tissues, cell lines	oncogene	EMT	proliferation, apoptosis, migration, invasion	55
CRNDE	RCC tissues, cell lines	oncogene	Wnt/β-catenin signaling	proliferation, growth, cell cycle	56
uc009yby.1	RCC tissues	oncogene		proliferation	57
FTX	RCC tissues, cell lines	oncogene		proliferation, cell cycle, migration, invasion	58
PVT1	RCC tissues	oncogene	MYC	promoter hypomethylation	59

**Table 2B:** Downregulated lncRNAs in RCC

lncRNAs	Specimens	Functions	Target genes/Signalings	Pathways/Mechanisms involved	Referances
CADM1-AS1	RCC tissues	tumor suppressor	CADM1	cell proliferation, apoptosis and migration	60
TRIM52-AS1	RCC tissues	tumor suppressor		proliferation, cell migration and apoptosis	61
GAS-5	RCC tissues	tumor suppressor		proliferation, apoptosis, cell cycle, migration, invasion	62
MEG3	RCC tissues, cell lines	tumor suppressor	Bcl-2, rocaspase-9, leaved caspase-9, cytochrome c	apoptosis, mitochondrial pathway	63
BX357664	RCC tissues, cell lines	tumor suppressor	EMT, MMP2, MMP9, TGF-β1/p38/HSP27	proliferation, migration, invasion, cell cycle	64
TCL6	RCC tissues	tumor suppressor		proliferation, apoptosis	65
CASC2	RCC tissues, cell lines	tumor suppressor	miR-21	proliferation, migration	66
NBAT-1	RCC tissues, cell lines	prognostic biomarker		proliferation, migration, invasion	67

The lncRNAs aberrantly expressed maybe participate in the carcinogenesis and progression of RCC. In addition, lncRNA profiling and the research results indicate that lncRNAs may be suitable for diagnostic, even in the prediction of prognostic purposes for RCC. A deeper study of these lncRNAs may pave a way for a further understanding in the basic science of RCC. Despite numerous efforts have been made, the mechanisms of lncRNAs, such as the transduction pathways and regulation functions of lncRNAs in RCC still need to be explored in further detail.

### Diagnosis and biomarkers

Many studies of determining aberrant lncRNAs expression for diagnostic purpose and the identification of novel deregulated lncRNAs as biomarkers for RCC have been carried out. A recent study found that the expression level of CYP4A22-2/3 can discriminate ccRCCs from normal kidney tissues [68]. In another study, lncRNA-ATB was found to be elevated in metastatic RCC patients, and higher expression of lncRNA-ATB correlated with disease progression and a more invasive feature, as well as metastasis [48]. In addition, RCCRT1 has a significant relationship with clinicopathologic features of ccRCC patients, including the size of tumor, the pathological staging and tumor grade [49], it is also associated with metastasis of lymph node as well as distal metastasis in ccRCC [49]. Moreover, TCL6 expression was an independent predictor of ccRCC aggressiveness and was negatively correlated with the T, N, M, and TNM stage [65].

The technique for detection and measurement of specific serum lncRNAs has become feasible, which provides a new method without invasiveness for the identification of new biomarker for cancer. Recent studies found that serum lncRNAs are useful for the diagnosis of multiple types of cancer, such as bladder cancer [69], liver cancer [70], cervical cancer [71], gastric cancer [72], colorectal cancer [73, 74] and breast cancer [75] etc, these serum lncRNAs may even serve as prognostic biomarkers [69, 72, 74]. Serum lncRNAs in RCC patients are also deregulated. Wu *et al* performed a study that demonstrated that five lncRNAs are significantly dysregulated in the serum of RCC patients-lncRNAs (LET, PVT1, PANDAR, PTENP1, and linc00963)- this 5 lncRNAs serum signature may obtain a preferable sensitivity and specificity for distinguishing ccRCC patients from normal individuals [76].

### Prognosis

A multitude of studies have shown that aberrant lncRNA expressions are associated with the disease overall survival (OS), the 5-year survival, the disease-free survival (DFS), the disease grade and stage, recurrence, metastasis etc. As shown in Table 3, decreased expression of lncRNA NONHSAT123350, CADM1-AS1, TCL6 and lnc-ZNF180-2 is correlated with poorer prognosis of RCC patients [38, 60, 65, 68]. On the other hand, overexpression of SPRY4-IT1, RCCRT1, MALAT1, Linc00152 and PVT1 shows a poor prognosis, as well [44, 49–52, 59].

**Table 3:** Prognostic lncRNAs in RCC

lncRNAs	Prognostic information	Referances
CADM1-AS1	decreased expression, poor prognosis (OS)	60
TCL6	low expression, poor prognosis (OS)	65
NONHSAT123350	low expression, poor prognosis (DFS, OS)	38
lnc-ZNF180-2	low level, poor progression-free, CSS and OS	68
SPRY4-IT1	high expression, poor prognosis (OS)	44
RCCRT1	high expression, poor survival	49
MALAT1	high expression, poor prognosis (OS)	50
Linc00152	high expression, poor prognosis (OS)	52
DRAIC	overexpression, good prognosis	77
PVT1	high expression, poor prognosis (OS)	59

The expression level of lncRNA NONHSAT123350 was closely associated with OS and DFS in patients without distant metastasis; the median values of OS and DFS were significantly higher among patients with high NONHSAT123350 expression when compared to the patients whose expression of NONHSAT123350 were low [38]. In another study, Ellinger *et al* reported the expression of lnc-ZNF180-2 significantly increased in advanced ccRCC tissue compared with localized ccRCC and normal renal tissue; a shorter period of progression-free survival (PFS), cancer-specific survival (CSS), and OS following nephrectomy happened in patients with lower levels of lnc-ZNF180-2 in the univariate Cox regression analysis. Thereafter, lnc-ZNF180-2 expression levels showed an independent predictor of progression-free survival (PSF), CSS, and OS in ccRCC patients within the multivariate Cox regression model [68]. In addition, the ccRCC patients had an advanced clinical stage and poorer prognosis when the SPRY4-IT1 expression was at high level. It is identified that SPRY4-IT1 is an independent prognostic factor in ccRCC in Cox proportional hazard model [44]. DRAIC is a new tumor suppressive sequence which locates on chromosome 15q23. DRAIC lncRNA was identified downregulated as prostate cancer cells progress from an androgen dependent to castration resistant state. DRAIC overexpression indicates a favorable prognosis in many kinds of malignancies including RCC [77]. Recently, a study showed that high Linc00152 expression was closely associated with advanced TNM stage in RCC patients. Moreover, Linc00152 is able to serve as an independent predictor of OS for the patients [52]. In addition, eight lncRNAs transcribed from the loci (ACTN4, CSNK1D, DNAJC3, GIGYF2, HDAC5, PTPN3, RAB25, and VPS13B) were detected as altered in the expression profiles in both of the malignancy and the survival outcomes [42]. Furthermore, PVT1 was strongly overexpressed in ccRCC and associated with the enhancement of MYC signaling and poorer clinical outcome [57].

### Therapy

Good ways to study the potential therapeutic function in cancer include lncRNAs are through loss-of-function or gain-of-function, RNA interfering technology, genetic loss-of-function models, or overexpression [33]. Therapeutic silencing or overexpression of these lncRNAs might be a viable therapeutic option to reduce the growth and/or metastatic potential of RCC. LncRNAs that may serve as therapeutic targets for RCC are listed in Table 4 and discussed as below.

**Table 4:** LncRNAs potentially serve as therapeutic targets for RCC

LncRNAs	Location	Properties/Mechanisms	References
HOTAIR	chromosome 12	recruit and bind on the locus of EZH2 and H3K27me3	80
		inhibit cycle-related genes p53, p21 and p16	80
		modulate covalent histones	80
		interact with methyltransferase PRC2, histone demethylase LSD1	80
		regulates gene silencing	80
		required for H3K27 trimethylation	81
		transcriptional silencing across the HOXD locus	81
RCCRT1	chr5:137801181-137805004	upregulated in RCC	49
		upregulated predicts poor survival of RCC	49
MALAT1	chromosome 11q13	independent predictor of OS in ccRCC	50, 51
		sequester serine/arginine splicing factors in nuclear speckle domains	83, 84
		regulate alternative splicing	84
		transcriptional activation of MALAT1 by c-Fos contribute to oncogenesis	51
		interact with Ezh2	51
		reciprocally repressed with miR205	51
		regulate EMT via E-cadherin and β-catenin	51
		promote ZEB2 expression by sponging miR-200s	85
H19	11p15.5	exon 1 of H19 harbors a miRNA-containing hairpin	86
		serve as the template of two miR6755p and miR6753p	86
LncRNA-SRLR	3q24	upregulated in intrinsically sorafenib resistant RCC	87
LncARSR	9q82.120.717-82.185.824	promote sunitinib resistance of RCC	88
		competitively bind to miR-34/miR-449	88
		facilitate AXL and c-MET expression	88
		exosome-transferred lncARSR confer sunitinib resistance	88
		targeting lncARSR restores sunitinib response in RCC	88
CADM1-AS1	antisense direction of a coding exon ofthe cell adhesion molecule 1 (CADM1)	involved in renal carcinogenesisindependent prognostic factor for ccRCC	60 60

#### HOTAIR

HOTAIR is transcribed from antisense strand of HOXC gene cluster present in chromosome 12 with a length of 2.2kb [78]. Recent study find that it can regulate EMT and is involved in Notch pathway [79]. Moreover, HOTAIR is a target of miR-141, inhibiting HOTAIR function in an Ago2-dependent manner [46]. Through RNAi technology, HOTAIR knockdown can affect cell cycle in G0/G1 phase and decrease cell proliferation and invasion of RCC cells [45]. The inhibiton of HOTAIR also suppressed tumor formation in the xenograft experiments *in vivo* [45]. Further studies are urgent for determining the regulatory mechanisms of HOTAIR, together with the interactions with epigenetic processes, such as DNA methylation, histone modification, microRNA and ubiquitin, by which new therapeutic targets might be developed in the treatment of RCC.

#### RCCRT1

RCCRT1 is upregulated in RCC compared with tumor adjacent tissue, particularly in high-grade RCC tissue. It is considered to be an oncogene of RCC, and elevated expression of RCCRT1 may predict dismal survival of RCC patients [49]. The knockdown of RCCRT1 by RNAi technique can suppress migration and invasion in RCC cell lines [49].

#### MALAT1

MALAT1 also called nuclear-enriched abundant transcript 2 (NEAT2) or Mhrt, it is one of the up-regulated nuclear lncRNAs in mammals [82]. The expression of MALAT1 is found to be upregulated in RCC tissues compared with corresponding normal tissues [50, 51]. Over-expression of MALAT1 indicates a more aggressive feature of the tumor and predicts a poorer OS than down-expression of MALAT1 in patients with ccRCC [50, 51]. *In vitro* experiments found that the inhibition of MALAT1 not only suppressed cell proliferation, promoted apoptosis, but also inhibited migration, and invasion of RCC cells [50, 51]. Thus, inhibition of MALAT1 may become a promising strategy for RCC therapy.

#### H19

The lncRNA H19 is another well-known oncogene in various cancer types, tumorigenesis, and cancer progression. It is transcribed from the sequence localized at 11p15.5 of the human genome. It is associate with disease progression and poorer prognosis of RCC patients [54]. Moreover, inhibition of H19 can suppress proliferation, invasion, and migration of RCC cells [54].

#### CADM1-AS1

LncRNA CADM1-AS is considered to be involved in the carcinogenesis of RCC. It is found to be decreased in RCC tissues [60]. Down-regulation of CADM1-AS1 correlates with advanced disease staging and poor prognosis of patients with ccRCC [60]. Over-expression of CADM1-AS1 can significantly decrease cell growth and migration, as well as increase apoptosis in RCC cells [60]. Therefore, CADMA1-AS1 maybe become a suitable therapeutic marker for RCC.

#### LncRNA-SRLR and lncARSR

Targeted therapy drugs, such as sorafenib and sunitinib, appear to increase the survival rate of RCC patients. But targeted therapy resistance is a major obstacle for the treatment of advanced and/or metastatic RCC. LncRNA-SRLR (sorafenib resistance-associated lncRNA in RCC) was found upregulated in intrinsically sorafenib resistant RCCs. Interestingly, lncRNA-SRLR knockdown sensitized nonresponsive RCC cells to sorafenib treatment, whereas the overexpression of lncRNA-SRLR conferred sorafenib resistance to responsive RCC cells. The underlying mechanism is that lncRNA-SRLR directly binds to NF-κB and promotes IL-6 transcription, leading to the activation of STAT3 and the development of sorafenib tolerance. A STAT3 inhibitor and IL-6-receptor antagonist both restored the response to sorafenib treatment. Moreover, high levels of lncRNA-SRLR correlated with poor responses to sorafenib therapy in RCC patients [87]. LncARSR (activated in RCC with sunitinib resistance, ENST00000424980) was a newly identified lncRNA to promote the sunitinib resistance of RCC. It is upregulated in sunitinib resistant RCC. By targeting lncARSR and the underlining pathways involved, sunitinib resistance RCC can restore the sensitivity to sunitinib [88]. Another study showed that lncARSR was upregulated in primary renal cancer stem cells (CSCs) and was associated with a poor prognosis of ccRCC. Knockdown of lncARSR attenuated the self-renewal, tumorigenicity, and metastasis of renal CSCs. Conversely, forced lncARSR expression enhanced CSC properties of RCC cells. The mechanism showed that the binding of lncARSR to Yes-associated protein (YAP) impedes LATS1-induced YAP phosphorylation and facilitates YAP nuclear translocation [89].

These findings are extremely meaningful, because lncRNAs may act as predictors and potential promising therapeutic targets for target therapy resistance, and may further improve the management of RCC patients receiving target therapy. If these lncRNAs could be applied to the treatment of RCC, it would benefit the patients, not only on efficacy, but also economically. Hence, further studies should be made for the identification of lncRNAs as targeted therapy biomarkers and application for RCC treatment strategy.

### Specific molecular mechanism of lncrnas in RCC

Hypoxia-inducible factor-2α (HIF2A) signaling participates in the RCC oncogenesis and progression as reported [90]. In this pathway, LncRNA-suppressing androgen receptor in renal cell carcinoma (SARCC) serves as a critical regulator, it connects with the AR/HIF2A/MYC axis to regulate cell growth in response to hypoxia. LncRNA-SARCC suppresses hypoxic cell cycle progression in VHL mutant type of RCC cells and de-represses it in VHL restored type of RCC cells. Mechanistically, lncRNA-SARCC binds to and deactivates androgen receptor (AR) protein to modulate AR post transcription thus block the AR/HIF2A/ MYC signals; on the other hand, HIF2A is able to regulate the lncRNA-SARCC expression on the transcription level by binding to hypoxia responsive elements on the lncRNA-SARCC promoter vice versa [90]. There is a negative feedback regulation loop between complex of LncRNA-SARCC/AR and HIF2A pathway, by which may result a differentially regulated RCC disease progression in a VHL dependent way [90]. Thereafter, future studies should be done to identify the association of lncRNAs and HIF signals in RCC.

Interestingly, a recent study from China established a novel method designed to identify lncRNA competitively regulated signal subpathways, so called subpathway-LNCE, which can identify lncRNA competitively regulated functions. The functional roles of these competitive regulation lncRNAs have not be well characterized in diseases. Moreover, the method integrated lncRNA-mRNA expression profile and pathway topologies [91].

## CONCLUSIONS

In conclusion, lncRNAs are aberrantly expressed in RCC, and participate in important roles in the regulation of RCC. They have a significant impact on our understanding the mechanisms of pathogenesis, progression and metastasis of RCC. They are also involve in key pathological process in RCC, the cell proliferation, cell cycle control, apoptosis, local invasion and distal metastasis. To deeply identify the mechanisms regarding how specific lncRNAs affect RCC and the regulatory mechanisms, further exploration and continued studies are required. At the same time, the lncRNA transduction pathway in RCC still needs to be further explored. Moreover, lncRNAs may act as promising new biomarkers for RCC, with the capability of improving diagnosis, predicting prognosis, and even in the improvement of therapeutic strategy for RCC patients. Several newly identified lncRNAs have the function of predicting target therapy resistance, by targeting these lncRNAs, the resistance of target therapies may be restored their sensitivity. RCC is an immunotherapy effective disease and the determination of whether lncRNAs could regulate the immune pathways of RCC such as PD-1 or PD-L1 signaling are expected. In any cases, to deeply understand the mechanisms of lncRNAs in RCC can not only help to uncover the tumorigenesis, but also have the possibility of application in the clinical management.
